# High-Frequency Vibration Analysis of Piezoelectric Array Sensor under Lateral-Field-Excitation Based on Crystals with 3 m Point Group

**DOI:** 10.3390/s22093596

**Published:** 2022-05-09

**Authors:** Jiachao Xu, Hao Shi, Fei Sun, Zehuan Tang, Shuanghuizhi Li, Dudu Chen, Tingfeng Ma, Iren Kuznetsova, Ilya Nedospasov, Chao Zhang

**Affiliations:** 1 Piezoelectric Device Laboratory, School of Mechanical Engineering and Mechanics, Ningbo University, Ningbo 315211, China; 1911081015@nbu.edu.cn (J.X.); shihao1990@nbu.edu.cn (H.S.); 2011081153@nbu.edu.cn (F.S.); 2011081009@nbu.edu.cn (Z.T.); 2111081008@nbu.edu.cn (S.L.); 1911081041@nbu.edu.cn (D.C.); 2 Kotelnikov Institute of Radio Engineering and Electronics of RAS, Moscow 125009, Russia; kuziren@yandex.ru (I.K.); ianedospasov@mail.ru (I.N.); 3 Research Institute of Tsinghua University in Shenzhen, Shenzhen 518057, China; zhangchao1969@tsinghua-sz.org

**Keywords:** bulk acoustic wave sensor, array devices, 3 m point group crystals, lateral-field-excitation, energy trapping

## Abstract

Based on Mindlin’s first-order plate theory, the high-frequency vibrations of piezoelectric bulk acoustic wave array sensors under lateral-field-excitation based on crystals with 3 m point group are analyzed, and the spectral-frequency relationships are solved, based on which, the optimal length–thickness ratio of the piezoelectric crystal plate is determined. Then, the dynamic capacitance diagram is obtained by a forced vibration analysis of the piezoelectric crystal plate. The resonant mode conforming to good energy trapping is further obtained. The frequency interferences between different resonator units are calculated, and the influences of the spacing between two resonant units on the frequency interference with different electrode widths and spacings are analyzed. Finally, the safe spacings between resonator units are obtained. As the electrode spacing value of the left unit increases, the safe spacing *d*_0_ between the two resonator units decreases, and the frequency interference curve tends to zero faster. When the electrode spacings of two resonator units are equal, the safe distance is largest, and the frequency interference curve tends to zero slowest. The theoretical results are verified further by finite element method. The analysis model of high frequency vibrations of strongly coupled piezoelectric bulk acoustic array device based on LiTaO_3_ crystals with 3 m point group proposed in this paper can provide reliable theoretical guidance for size optimization designs of strongly coupled piezoelectric array sensors under lateral-field-excitation.

## 1. Introduction

The traditional electric field excitation mode for piezoelectric bulk acoustic wave devices is thickness-field-excitation (TEF) [1,2,3,4,5,6], for which the electrodes are arranged on the upper and lower surfaces of the crystal plate, and the direction of the electric field is along the thickness direction of the crystal plate. Later, it was found that bulk acoustic devices can also operate in lateral-field-excitation (LFE) mode. Initially, the electrodes of LFE devices were arranged on either side surface of the crystal plate [7]. However, because the crystal plate is too thin, it is difficult to place electrodes on the side surface. In recent years, an effective method emerges, namely, the electrodes of LFE devices were placed on the same surface (top or bottom surface) of the crystal plate [8,9,10,11,12], and the direction of the electric field is perpendicular to the thickness direction of the crystal plate. LFE devices have the following advantages: because of the electrode arrangement, it is easier to package the device; the unnecessary vibration modes can be eliminated by changing the orientation of the electrodes; there is only weak vibration in the middle unelectroded region, which reduces the aging rate of the device [9,10,11].

For piezoelectric bulk acoustic wave sensors with a single resonator unit, the measurement accuracy is influenced by the ambient temperature and humidity [13]. In addition, in biological detection and mixed gas composition analysis, the bulk acoustic wave sensor with a single resonant unit cannot measure multiple components simultaneously [14]. In recent years, piezoelectric crystal microbalance array emerged. For this type of device, there are several resonator units made on a single crystal plate [15,16,17], among which, a reference unit can be set to eliminate the influence of environmental factors. Different selective adsorption films can be made on different units to achieve simultaneous measurement of multiple components [16,17].

Piezoelectric bulk acoustic wave array devices excited by lateral electric fields have a good application prospect in multi-component sensing. At present, quartz crystal is usually the crystal plate material used in LFE piezoelectric bulk acoustic wave array device, but the quartz crystal has low piezoelectric coupling coefficients, and is difficult to meet the requirements of measurement with high precision, high sensitivity, and large damping [18]. Cubic 3 m point group piezoelectric crystals (LiTaO_3_, LiNbO_3_, etc.) have high piezoelectric coupling coefficients [19,20], thus LFE bulk acoustic wave devices based on such crystal materials have obvious advantages. However, due to its high piezoelectric coupling coefficient, the electric field and displacement distributions of strongly coupled LFE array devices are more complex than that base on quartz crystals, and the energy trapping characteristics of the device are still not clear. In addition, the frequency interferences between adjacent units are obvious, the effect of the structure parameters on which need to be clarified.

In this paper, the high-frequency vibration analysis model of strongly coupled piezoelectric bulk acoustic devices based on 3 m point group crystals excited by lateral electric fields is established, the coupling relationships between vibration modes are clarified, and the influence of structural parameters on the frequency interference between resonator units are revealed, which provides reliable guidance for the size design of strongly coupled LFE array devices based crystals with 3 m point group. The mathematical model in this work is based on Mindlin’s first-order plate theory, which is an approximate two-dimensional theory. The calculation error of the frequency shift is negligible [7]. Compared with the finite element method, the method based on Mindlin’s first-order plate theory could clarify the mechanisms of frequency interferences between different resonator units conveniently, and the calculation time is shortened obviously.

## 2. Frequency Spectrum Calculation

**Figure 1:**
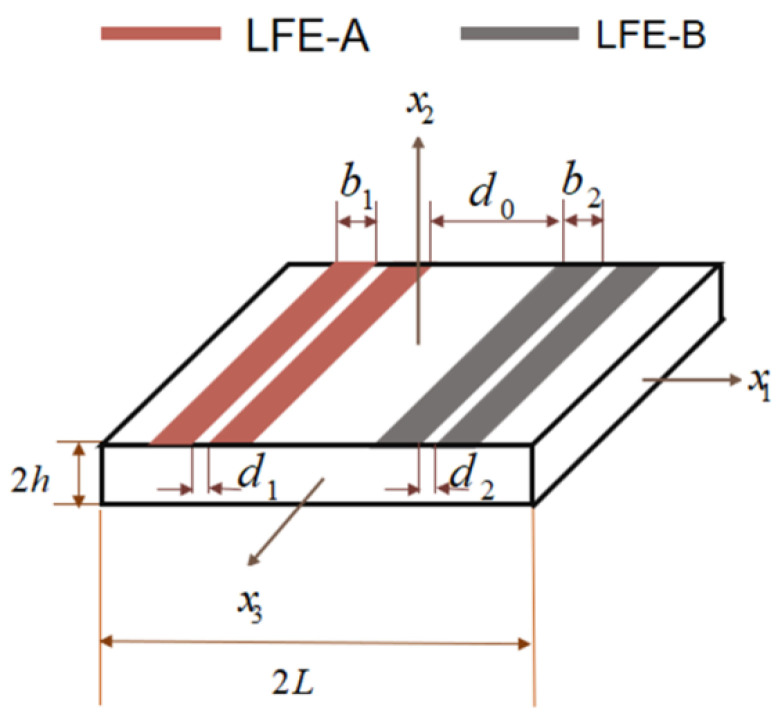
Structure diagram of LiTaO_3_ LFE bulk acoustic wave array devices.

Figure 1 shows the structure diagram of LiTaO_3_ LFE bulk acoustic wave array devices. Two pairs of electrodes are placed in the top surface of the crystal plate, forming two resonator units RU-A and RU-B. b_1_ and b_2_ are the electrode widths of RU-A and RU-B, respectively. *d*_1_ and *d*_2_ are the electrode spacings of RU-A and RU-B, respectively. *d*_0_ is the spacing between the two resonator units; 2 *L* and 2 *h* are the length and thickness of the crystal plate. $\rho_{e}$ and $2h_{e}$ are the density and thickness of the electrodes, respectively.

For 3 m point group piezoelectric single crystal, the motion control equation of unelectroded region is:

(1) $$\begin{array}{cc} \; & k_{1}C_{65}u_{3,11}^{\left(0\right)}+k_{1}^{2}C_{66}u_{2,11}^{\left(0\right)}+k_{1}^{2}C_{66}u_{1,1}^{\left(1\right)}+k_{1}e_{16}\phi_{,11}^{\left(0\right)}=\rho {\ddot{u}}_{2}^{\left(0\right)},\\ \; & C_{55}u_{3,11}^{\left(0\right)}+k_{1}C_{56}u_{2,11}^{\left(0\right)}+k_{1}C_{56}u_{1,1}^{\left(1\right)}+e_{15}\phi_{,11}^{\left(0\right)}=\rho {\ddot{u}}_{3}^{\left(0\right)},\\ \; & \gamma_{11}u_{1,11}^{\left(1\right)}-3h^{-2}\left[k_{1}C_{65}u_{3,1}^{\left(0\right)}+k_{1}^{2}C_{66}u_{2,1}^{\left(0\right)}+k_{1}^{2}C_{66}u_{1}^{\left(1\right)}+k_{1}e_{16}\phi_{,1}^{\left(0\right)}\right]=\rho {\ddot{u}}_{1}^{\left(1\right)},\\ \; & e_{15}u_{3,11}^{\left(0\right)}+k_{1}e_{16}\left(u_{2,11}^{\left(0\right)}+u_{1,1}^{\left(1\right)}\right)-\varepsilon_{11}\phi_{,11}^{\left(0\right)}=0, \end{array}$$

Where $c_{pq}(=c_{pq}^{E})$, $e_{ip}$ and $\varepsilon_{ij}\left(=\varepsilon_{ij}^{S}\right)$ are elastic stiffness, piezoelectric constant, and dielectric constant, respectively.

The motion control equation of electroded region is:

(2) $$\begin{array}{l} {\bar{k}}_{1}C_{65}u_{3,11}^{\left(0\right)}+{\bar{k}}_{1}^{2}C_{66}u_{2,11}^{\left(0\right)}+{\bar{k}}_{1}^{2}C_{66}u_{1,1}^{\left(1\right)}=\left(1+R\right)\rho {\ddot{u}}_{2}^{\left(0\right)},\\ C_{55}u_{3,11}^{\left(0\right)}+{\bar{k}}_{1}C_{56}u_{2,11}^{\left(0\right)}+{\bar{k}}_{1}C_{56}u_{1,1}^{\left(1\right)}=\left(1+R\right)\rho {\ddot{u}}_{3}^{\left(0\right)},\\ \gamma_{11}u_{1,11}^{\left(1\right)}-3h^{-2}\left[{\bar{k}}_{1}C_{65}u_{3,1}^{\left(0\right)}+{\bar{k}}_{1}^{2}C_{66}u_{2,1}^{\left(0\right)}+{\bar{k}}_{1}^{2}C_{66}u_{1}^{\left(1\right)}\right]=\left(1+3R\right)\rho {\ddot{u}}_{1}^{\left(1\right)}. \end{array}$$

According to the standing wave hypothesis of the finite plate, the forms of displacement and potential are assumed to be:

(3) $$\begin{array}{l} u_{2}^{\left(0\right)}=A_{1}\sin\left(\xi x_{1}\right)e^{i\omega t},\\ u_{3}^{\left(0\right)}=A_{2}\sin\left(\xi x_{1}\right)e^{i\omega t},\\ u_{1}^{\left(1\right)}=A_{3}\cos\left(\xi x_{1}\right)e^{i\omega t},\\ \phi^{\left(0\right)}=A_{4}\sin\left(\xi x_{1}\right)e^{i\omega t}. \end{array}$$

By substituting (3) into the governing Equation (1), a set of four-order linear equations for amplitudes *A*_1_−*A*_4_ is obtained. Since the amplitudes has non-zero solutions, the determinant of its coefficient matrix is zero, and a four-order polynomial about the wave number can be obtained. Finally, four corresponding solutions are obtained by solving this polynomial, including three non-zero solutions and one zero solution.

When ${\left(\xi^{\left(m\right)}\right)}^{2}=0$, $\phi_{,1}^{\left(0\right)}=-Ee^{i\omega t}$ is assumed to be the form of electric field excitation, and *E* is the voltage of excitation. $\phi^{\left(0\right)}$ is a linear function about coordinate *x*_1_, namely $\phi^{\left(0\right)}=\phi^{\left(0\right)}\left(x_{1}\right)$. After substituting that into Equation (3), displacements and electric potential can be obtained in the form:

(4) $$\begin{array}{l} u_{2}^{\left(0\right)}=0,\\ u_{3}^{\left(0\right)}=0,\\ u_{1}^{\left(1\right)}=A_{3}e^{i\omega t},\\ \phi^{\left(0\right)}=\phi^{\left(0\right)}\left(x_{1}\right). \end{array}$$

Substituting (4) into (1), we obtain:

(5) $$-3h^{-2}\left[k_{1}^{2}C_{66}u_{1}^{\left(1\right)}+k_{1}e_{16}\phi_{,1}^{\left(0\right)}\right]=\rho {\ddot{u}}_{1}^{\left(1\right)},$$

where $u_{1}^{\left(1\right)}=-B_{1}Ee^{i\omega t}.$ By solving Equation (5), we obtain:

(6) $$B_{1}=\frac{-k_{1}e_{16}}{k_{1}^{2}C_{66}-\frac{\pi^{2}}{12}C_{66}\Omega^{2}}.$$

Based on the above equation, the forms of displacement and potential solution are set as follows:

(7) $$\left\{\begin{array}{c} u_{2}^{\left(0\right)}\\ u_{3}^{\left(0\right)}\\ u_{1}^{\left(1\right)}\\ \phi^{\left(0\right)} \end{array}\right\}=\sum_{m=1}^{3}c^{\left(m\right)}\left\{\begin{array}{c} \beta_{1}^{\left(m\right)}\sin\left(\xi^{\left(m\right)}x_{1}\right)\\ \beta_{2}^{\left(m\right)}\sin\left(\xi^{\left(m\right)}x_{1}\right)\\ \beta_{3}^{\left(m\right)}\cos\left(\xi^{\left(m\right)}x_{1}\right)\\ \beta_{4}^{\left(m\right)}\sin\left(\xi^{\left(m\right)}x_{1}\right) \end{array}\right\}+c^{\left(4\right)}\left\{\begin{array}{c} 0\\ 0\\ B_{1}\\ x_{1} \end{array}\right\},$$

where $\beta_{i}^{\left(m\right)}$ is the amplitude ratio, namely $\frac{A_{ri}}{A_{4i}}\left(r=1-4,i=1-4\right)$, which can be determined by

(8) $$\left[\begin{array}{ccc} C_{66}\Omega^{2}-k_{1}^{2}C_{66}{Z_{i}}^{2} & -k_{1}C_{65}{Z_{i}}^{2} & -\frac{2h}{\pi }k_{1}^{2}C_{66}Z_{i}\\ -k_{1}C_{56}{Z_{i}}^{2} & C_{66}\Omega^{2}-C_{55}{Z_{i}}^{2} & -\frac{2h}{\pi }k_{1}C_{56}Z_{i}\\ -\frac{6}{\pi h}k_{1}^{2}C_{66}{Z_{i}}^{2} & -\frac{6}{\pi h}k_{1}C_{65}{Z_{i}}^{2} & C_{66}\Omega^{2}-\gamma_{11}{Z_{i}}^{2}-\frac{12}{\pi^{2}}k_{1}^{2}C_{66} \end{array}\right]\left[\begin{array}{c} \frac{A_{1i}}{A_{4i}}\\ \frac{A_{2i}}{A_{4i}}\\ \frac{A_{3i}}{A_{4i}} \end{array}\right]=\left[\begin{array}{c} k_{1}e_{16}{Z_{i}}^{2}\\ e_{15}{Z_{i}}^{2}\\ -\frac{6}{\pi h}k_{1}e_{16}Z_{i} \end{array}\right].$$

Boundary conditions are as follows:

(9) $$T_{6}^{\left(0\right)}=0,T_{5}^{\left(0\right)}=0,T_{1}^{\left(1\right)}=0,D_{1}^{\left(0\right)}=0,x_{1}=\pm L.$$

Substituting Equation (7) into Equation (9), we obtain

(10) $$\left|\begin{array}{cccc} H_{1}\cos\left(\frac{\pi Z_{1}}{2}\frac{c}{h}\right) & H_{2}\cos\left(\frac{\pi Z_{2}}{2}\frac{c}{h}\right) & H_{3}\cos\left(\frac{\pi Z_{3}}{2}\frac{c}{h}\right) & H_{4}\\ I_{1}\sin\left(\frac{\pi Z_{1}}{2}\frac{c}{h}\right) & I_{2}\sin\left(\frac{\pi Z_{2}}{2}\frac{c}{h}\right) & I_{3}\sin\left(\frac{\pi Z_{3}}{2}\frac{c}{h}\right) & 0\\ \beta_{3}^{\left(1\right)}\sin\left(\frac{\pi Z_{1}}{2}\frac{c}{h}\right) & \beta_{3}^{\left(2\right)}\sin\left(\frac{\pi Z_{2}}{2}\frac{c}{h}\right) & \beta_{3}^{\left(3\right)}\sin\left(\frac{\pi Z_{3}}{2}\frac{c}{h}\right) & x_{3}\\ \beta_{4}^{\left(1\right)}\cos\left(\frac{\pi Z_{1}}{2}\frac{c}{h}\right) & \beta_{4}^{\left(2\right)}\cos\left(\frac{\pi Z_{2}}{2}\frac{c}{h}\right) & \beta_{4}^{\left(3\right)}\cos\left(\frac{\pi Z_{3}}{2}\frac{c}{h}\right) & B \end{array}\right|=0,$$

where

(11) $$\begin{array}{cc} \; & H_{i}=2h\left[k_{1}C_{65}\beta_{2}^{\left(i\right)}\frac{\pi Z_{i}}{2h}+k_{1}^{2}C_{66}\left(\beta_{1}^{\left(i\right)}\frac{\pi Z_{i}}{2h}+\beta_{3}^{\left(i\right)}\right)+k_{1}e_{16}\frac{\pi Z_{i}}{2h}\beta_{4}^{\left(i\right)}\right],\\ \; & H_{4}=2h\left(k_{1}^{2}C_{66}B_{1}+k_{1}e_{16}\right),\\ \; & I_{i}=2h\left[C_{55}\beta_{2}^{\left(i\right)}\frac{\pi Z_{i}}{2h}+k_{1}C_{56}\left(\beta_{1}^{\left(i\right)}\frac{\pi Z_{i}}{2h}+\beta_{3}^{\left(i\right)}\right)+e_{16}\frac{\pi Z_{i}}{2h}\beta_{4}^{\left(i\right)}\right],\\ \; & I_{4}=2h\left(k_{1}C_{56}B_{1}+e_{15}\right),\\ \; & J_{i}=\frac{2h^{3}}{3}\left[-\gamma_{11}\beta_{3}^{\left(i\right)}\frac{\pi Z_{i}}{2h}\right],\\ \; & W_{i}=2h\left[e_{15}\beta_{2}^{\left(i\right)}\frac{\pi Z_{i}}{2h}+k_{1}e_{16}\left(\beta_{1}^{\left(i\right)}\frac{\pi Z_{i}}{2h}+\beta_{3}^{\left(i\right)}\right)-\varepsilon_{11}\frac{\pi Z_{i}}{2h}\beta_{4}^{\left(i\right)}\right]\\ \; & W_{4}=2h\left(k_{1}e_{16}B_{1}-\xi_{11}\right). \end{array}$$

**Figure 2:**
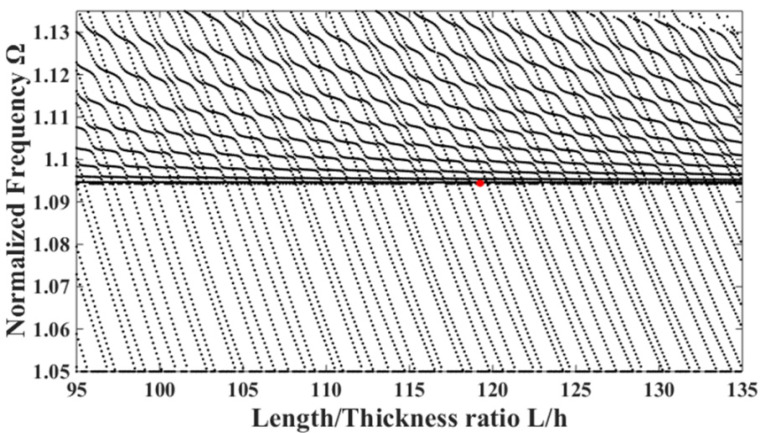
Relationship between the frequency and the ratios of the length to thickness of the LiTaO_3_ crystal plate excited by lateral electric fields.

By solving Equation (10), the spectrum diagram of the LiTaO_3_ crystal plate excited by lateral electric fields can be obtained, as shown in Figure 2.

In Figure 2, the horizontal line is the main vibration mode, namely the thickness- shear mode. The slanted curved lines in the upper part represent the bend modes, and the slanted straight lines represent the face-shear modes. In the figure, two types of curves will form an intersection point, which is with the strongest coupling between different modes. The middle point between two intersections is with the weakest coupling, such as the mode showed by the red point.

## 3. Electrically Forced Vibration

**Figure 3:**
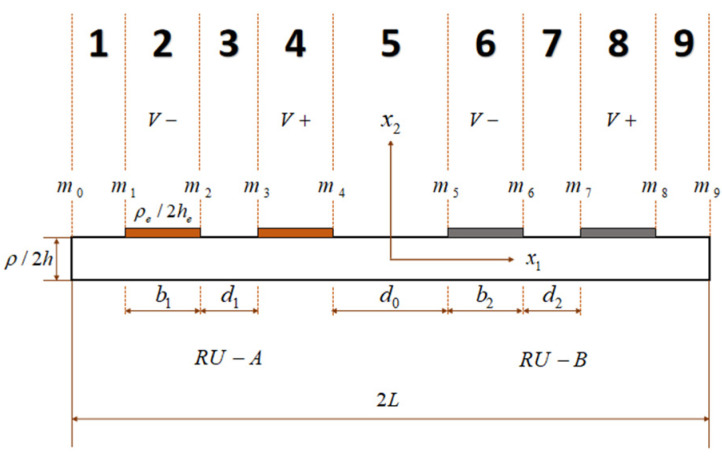
The partition diagram of LiTaO_3_ array devices with lateral-field-excitation.

As shown in Figure 3, the device is divided into 9 regions, 1 and 3 are the unelectroded regions of Ru-A, 2 and 4 are the electroded regions of Ru-A, 5 are the unelectroded region between the two units, 6 and 8 are the electroded regions of Ru-B, and 7 and 9 are the unelectroded regions of Ru-B. $m_{0}$ and $m_{9}$ are boundary points of the device. $m_{1}\sim m_{8}$ is the junction point of unelectroded and electroded regions of two resonator units.

The forms of the displacements and potential of the unelectroded regions are assumed to be

(12) $$\begin{array}{l} u_{2}^{\left(0\right)}=A_{1}e^{i\xi x_{1}}e^{i\omega t},\\ u_{3}^{\left(0\right)}=A_{2}e^{i\xi x_{1}}e^{i\omega t},\\ u_{1}^{\left(1\right)}=A_{3}e^{i\xi x_{1}}e^{i\omega t},\\ \phi^{\left(0\right)}=A_{4}e^{i\xi x_{1}}e^{i\omega t}. \end{array}$$

By substituting Equation (12) into the governing Equation (1), a set of fourth-order linear equations about amplitudes *A*_1_–*A*_4_ are obtained. Since the amplitude has non-zero solutions, the determinant of coefficient matrix of equations is zero, and a fourth-order polynomial about wave number can be obtained. Finally, eight frequency solutions corresponding to wave number are obtained by solving this polynomial, including six non-zero solutions and two zero solutions:

(13) $$\left\{\begin{array}{l} u_{2}^{\left(0\right)}\\ u_{3}^{\left(0\right)}\\ u_{1}^{\left(1\right)}\\ \phi^{\left(0\right)} \end{array}\right\}=\sum_{m=1}^{6}{\tilde{c}}^{\left(m\right)}\left\{\begin{array}{l} {\tilde{\beta }}_{1}^{\left(m\right)}e^{i\xi^{\left(m\right)}x_{1}}\\ {\tilde{\beta }}_{2}^{\left(m\right)}e^{i\xi^{\left(m\right)}x_{1}}\\ {\tilde{\beta }}_{3}^{\left(m\right)}e^{i\xi^{\left(m\right)}x_{1}}\\ {\tilde{\beta }}_{4}^{\left(m\right)}e^{i\xi^{\left(m\right)}x_{1}} \end{array}\right\}+{\tilde{c}}^{\left(7\right)}\left\{\begin{array}{l} 0\\ 0\\ {\tilde{B}}_{1}\\ x_{1} \end{array}\right\}+{\tilde{c}}^{\left(8\right)}\left\{\begin{array}{l} 0\\ 0\\ 0\\ 1 \end{array}\right\}$$

For unelectroded regions 3 and 7,

(14) $$B_{1}=\frac{-k_{1}e_{16}}{k_{1}^{2}C_{66}-\frac{\pi^{2}}{12}C_{66}\Omega^{2}}.$$

For unelectroded regions 1, 5, and 9, $B_{2}=-B_{1}$. $c^{\left(1\right)}-c^{\left(8\right)}$ are undetermined constants, ${\tilde{\beta }}_{i}^{\left(m\right)}$ is the amplitude ratio, namely $\frac{A_{ri}}{A_{4i}}\left(r=1-4,i=1-8\right)$, which can be determined by

(15) $$\left[\begin{array}{ccc} C_{66}\Omega^{2}-k_{1}^{2}C_{66}Z_{j}^{2} & -k_{1}C_{65}Z_{j}^{2} & i\frac{2h}{\pi }k_{1}^{2}C_{66}Z_{j}\\ -k_{1}C_{56}Z_{j}^{2} & C_{66}\Omega^{2}-C_{55}Z_{j}^{2} & i\frac{2h}{\pi }k_{1}C_{56}Z_{j}\\ -i\frac{6}{\pi h}k_{1}^{2}C_{66}Z_{j} & -i\frac{6}{\pi h}k_{1}C_{65}Z_{j} & C_{66}\Omega^{2}-\gamma_{11}Z_{j}^{2}-\frac{12}{\pi^{2}}k_{1}^{2}C_{66} \end{array}\right]\left[\begin{array}{c} {\tilde{\beta }}_{1}^{\left(m\right)}\\ {\tilde{\beta }}_{2}^{\left(m\right)}\\ {\tilde{\beta }}_{3}^{\left(m\right)} \end{array}\right]=\left[\begin{array}{c} k_{1}e_{16}Z_{j}^{2}\\ e_{15}Z_{j}^{2}\\ i\frac{6}{\pi h}k_{1}e_{16}Z_{j} \end{array}\right]$$

The forms of the displacements and potential of the electroded regions are assumed to be

(16) $$\begin{array}{l} u_{2}^{\left(0\right)}=A_{1}e^{i\bar{\xi }x_{1}}e^{i\omega t},\\ u_{3}^{\left(0\right)}=A_{2}e^{i\bar{\xi }x_{1}}e^{i\omega t},\\ u_{1}^{\left(1\right)}=A_{3}e^{i\bar{\xi }x_{1}}e^{i\omega t}. \end{array}$$

Substituting (16) into (2), a set of third-order linear equations with respect to $A_{1}-A_{3}$ are obtained. When the determinant of the coefficient matrix of the equations is zero, non-zero solutions exist. Base on that we can obtain a third order polynomial with wave number. By solving the third-order polynomial, six wavenumber solutions are obtained, namely three pairs of non-zero conjugate solutions.

(17) $$\left\{\begin{array}{l} u_{2}^{\left(0\right)}\\ u_{3}^{\left(0\right)}\\ u_{1}^{\left(1\right)} \end{array}\right\}=\sum_{m=1}^{6}{\bar{C}}^{\left(m\right)}\left\{\begin{array}{l} {\bar{\beta }}_{1}^{\left(m\right)}e^{i{\bar{\xi }}^{\left(m\right)}x_{1}}\\ {\bar{\beta }}_{2}^{\left(m\right)}e^{i{\bar{\xi }}^{\left(m\right)}x_{1}}\\ {\bar{\beta }}_{3}^{\left(m\right)}e^{i{\bar{\xi }}^{\left(m\right)}x_{1}} \end{array}\right\},$$

where ${\bar{C}}^{\left(m\right)}\left(m=1-6\right)$ are undetermined constants, ${\bar{\beta }}_{i}^{\left(m\right)}$ is the amplitude ratio, which can be determined by

(18) $$\left[\begin{array}{cc} (1+R)C_{66}\Omega^{2}-{\bar{k}}_{1}^{2}C_{66}Z_{j}^{2} & -{\bar{k}}_{1}C_{65}Z_{j}^{2}\\ -{\bar{k}}_{1}C_{56}Z_{j}^{2} & (1+R)C_{66}\Omega^{2}-C_{55}Z_{j}^{2} \end{array}\right]\left[\begin{array}{c} {\bar{\beta }}_{1}^{\left(m\right)}\\ {\bar{\beta }}_{2}^{\left(m\right)} \end{array}\right]=\left[\begin{array}{c} -i\frac{2h}{\pi }{\bar{k}}_{1}^{2}C_{66}Z_{j}^{2}\\ -i\frac{2h}{\pi }{\bar{k}}_{1}C_{56}Z_{j}^{2} \end{array}\right].$$

For *m*_0_ and *m*_9_, boundary conditions are

(19) $$\begin{array}{l} T_{5}^{\left(0\right)}\left(x_{1}=m_{0}\right)=0,\\ T_{6}^{\left(0\right)}\left(x_{1}=m_{0}\right)=0,\\ T_{1}^{\left(1\right)}\left(x_{1}=m_{0}\right)=0,\\ D_{1}^{\left(0\right)}\left(x_{1}=m_{0}\right)=0. \end{array}$$

(20) $$\begin{array}{l} T_{5}^{\left(0\right)}\left(x_{1}=m_{9}\right)=0,\\ T_{6}^{\left(0\right)}\left(x_{1}=m_{9}\right)=0,\\ T_{1}^{\left(1\right)}\left(x_{1}=m_{9}\right)=0,\\ D_{1}^{\left(0\right)}\left(x_{1}=m_{9}\right)=0. \end{array}$$

For *m*_1_~*m*_8_, continuous conditions are

(21) $$\begin{array}{l} u_{2}^{\left(0\right)}\left(x_{1}=m_{1}^{-}\right)=u_{2}^{\left(0\right)}\left(x_{1}=m_{1}^{+}\right)\\ u_{3}^{\left(0\right)}\left(x_{1}=m_{1}^{-}\right)=u_{3}^{\left(0\right)}\left(x_{1}=m_{1}^{+}\right)\\ u_{1}^{\left(1\right)}\left(x_{1}=m_{1}^{-}\right)=u_{1}^{\left(1\right)}\left(x_{1}=m_{1}^{+}\right)\\ T_{5}^{\left(0\right)}\left(x_{1}=m_{1}^{-}\right)=T_{5}^{\left(0\right)}\left(x_{1}=m_{1}^{+}\right)\\ T_{6}^{\left(0\right)}\left(x_{1}=m_{1}^{-}\right)=T_{6}^{\left(0\right)}\left(x_{1}=m_{1}^{+}\right)\\ T_{1}^{\left(1\right)}\left(x_{1}=m_{1}^{-}\right)=T_{1}^{\left(1\right)}\left(x_{1}=m_{1}^{+}\right)\\ \phi^{\left(0\right)}\left(x_{1}=m_{1}^{-}\right)=-Ve^{i\omega t} \end{array}$$

(22) $$\begin{array}{l} u_{2}^{\left(0\right)}\left(x_{1}=m_{2}^{-}\right)=u_{2}^{\left(0\right)}\left(x_{1}=m_{2}^{+}\right)\\ u_{3}^{\left(0\right)}\left(x_{1}=m_{2}^{-}\right)=u_{3}^{\left(0\right)}\left(x_{1}=m_{2}^{+}\right)\\ u_{1}^{\left(1\right)}\left(x_{1}=m_{2}^{-}\right)=u_{1}^{\left(1\right)}\left(x_{1}=m_{2}^{+}\right)\\ T_{5}^{\left(0\right)}\left(x_{1}=m_{2}^{-}\right)=T_{5}^{\left(0\right)}\left(x_{1}=m_{2}^{+}\right)\\ T_{6}^{\left(0\right)}\left(x_{1}=m_{2}^{-}\right)=T_{6}^{\left(0\right)}\left(x_{1}=m_{2}^{+}\right)\\ T_{1}^{\left(1\right)}\left(x_{1}=m_{2}^{-}\right)=T_{1}^{\left(1\right)}\left(x_{1}=m_{2}^{+}\right)\\ \phi^{\left(0\right)}\left(x_{1}=m_{2}^{-}\right)=-Ve^{i\omega t} \end{array}$$

(23) $$\begin{array}{l} u_{2}^{\left(0\right)}\left(x_{1}=m_{3}^{-}\right)=u_{2}^{\left(0\right)}\left(x_{1}=m_{3}^{+}\right)\\ u_{3}^{\left(0\right)}\left(x_{1}=m_{3}^{-}\right)=u_{3}^{\left(0\right)}\left(x_{1}=m_{3}^{+}\right)\\ u_{1}^{\left(1\right)}\left(x_{1}=m_{3}^{-}\right)=u_{1}^{\left(1\right)}\left(x_{1}=m_{3}^{+}\right)\\ T_{5}^{\left(0\right)}\left(x_{1}=m_{3}^{-}\right)=T_{5}^{\left(0\right)}\left(x_{1}=m_{3}^{+}\right)\\ T_{6}^{\left(0\right)}\left(x_{1}=m_{3}^{-}\right)=T_{6}^{\left(0\right)}\left(x_{1}=m_{3}^{+}\right)\\ T_{1}^{\left(1\right)}\left(x_{1}=m_{3}^{-}\right)=T_{1}^{\left(1\right)}\left(x_{1}=m_{3}^{+}\right)\\ \phi^{\left(0\right)}\left(x_{1}=m_{3}^{-}\right)=Ve^{i\omega t} \end{array}$$

(24) $$\begin{array}{l} u_{2}^{\left(0\right)}\left(x_{1}=m_{4}^{-}\right)=u_{2}^{\left(0\right)}\left(x_{1}=m_{1}^{+}\right)\\ u_{3}^{\left(0\right)}\left(x_{1}=m_{4}^{-}\right)=u_{3}^{\left(0\right)}\left(x_{1}=m_{1}^{+}\right)\\ u_{1}^{\left(1\right)}\left(x_{1}=m_{4}^{-}\right)=u_{1}^{\left(1\right)}\left(x_{1}=m_{1}^{+}\right)\\ T_{5}^{\left(0\right)}\left(x_{1}=m_{4}^{-}\right)=T_{5}^{\left(0\right)}\left(x_{1}=m_{1}^{+}\right)\\ T_{6}^{\left(0\right)}\left(x_{1}=m_{4}^{-}\right)=T_{6}^{\left(0\right)}\left(x_{1}=m_{1}^{+}\right)\\ T_{1}^{\left(1\right)}\left(x_{1}=m_{4}^{-}\right)=T_{1}^{\left(1\right)}\left(x_{1}=m_{1}^{+}\right)\\ \phi^{\left(0\right)}\left(x_{1}=m_{4}^{-}\right)=Ve^{i\omega t} \end{array}$$

(25) $$\begin{array}{l} u_{2}^{\left(0\right)}\left(x_{1}=m_{5}^{-}\right)=u_{2}^{\left(0\right)}\left(x_{1}=m_{5}^{+}\right)\\ u_{3}^{\left(0\right)}\left(x_{1}=m_{5}^{-}\right)=u_{3}^{\left(0\right)}\left(x_{1}=m_{5}^{+}\right)\\ u_{1}^{\left(1\right)}\left(x_{1}=m_{5}^{-}\right)=u_{1}^{\left(1\right)}\left(x_{1}=m_{5}^{+}\right)\\ T_{5}^{\left(0\right)}\left(x_{1}=m_{5}^{-}\right)=T_{5}^{\left(0\right)}\left(x_{1}=m_{5}^{+}\right)\\ T_{6}^{\left(0\right)}\left(x_{1}=m_{5}^{-}\right)=T_{6}^{\left(0\right)}\left(x_{1}=m_{5}^{+}\right)\\ T_{1}^{\left(1\right)}\left(x_{1}=m_{5}^{-}\right)=T_{1}^{\left(1\right)}\left(x_{1}=m_{5}^{+}\right)\\ \phi^{\left(0\right)}\left(x_{1}=m_{5}^{-}\right)=-Ve^{i\omega t} \end{array}$$

(26) $$\begin{array}{l} u_{2}^{\left(0\right)}\left(x_{1}=m_{6}^{-}\right)=u_{2}^{\left(0\right)}\left(x_{1}=m_{6}^{+}\right)\\ u_{3}^{\left(0\right)}\left(x_{1}=m_{6}^{-}\right)=u_{3}^{\left(0\right)}\left(x_{1}=m_{6}^{+}\right)\\ u_{1}^{\left(1\right)}\left(x_{1}=m_{6}^{-}\right)=u_{1}^{\left(1\right)}\left(x_{1}=m_{6}^{+}\right)\\ T_{5}^{\left(0\right)}\left(x_{1}=m_{6}^{-}\right)=T_{5}^{\left(0\right)}\left(x_{1}=m_{6}^{+}\right)\\ T_{6}^{\left(0\right)}\left(x_{1}=m_{6}^{-}\right)=T_{6}^{\left(0\right)}\left(x_{1}=m_{6}^{+}\right)\\ T_{1}^{\left(1\right)}\left(x_{1}=m_{6}^{-}\right)=T_{1}^{\left(1\right)}\left(x_{1}=m_{6}^{+}\right)\\ \phi^{\left(0\right)}\left(x_{1}=m_{6}^{-}\right)=-Ve^{i\omega t} \end{array}$$

(27) $$\begin{array}{l} u_{2}^{\left(0\right)}\left(x_{1}=m_{7}^{-}\right)=u_{2}^{\left(0\right)}\left(x_{1}=m_{7}^{+}\right)\\ u_{3}^{\left(0\right)}\left(x_{1}=m_{7}^{-}\right)=u_{3}^{\left(0\right)}\left(x_{1}=m_{7}^{+}\right)\\ u_{1}^{\left(1\right)}\left(x_{1}=m_{7}^{-}\right)=u_{1}^{\left(1\right)}\left(x_{1}=m_{7}^{+}\right)\\ T_{5}^{\left(0\right)}\left(x_{1}=m_{7}^{-}\right)=T_{5}^{\left(0\right)}\left(x_{1}=m_{7}^{+}\right)\\ T_{6}^{\left(0\right)}\left(x_{1}=m_{7}^{-}\right)=T_{6}^{\left(0\right)}\left(x_{1}=m_{7}^{+}\right)\\ T_{1}^{\left(1\right)}\left(x_{1}=m_{7}^{-}\right)=T_{1}^{\left(1\right)}\left(x_{1}=m_{7}^{+}\right)\\ \phi^{\left(0\right)}\left(x_{1}=m_{7}^{-}\right)=Ve^{i\omega t} \end{array}$$

(28) $$\begin{array}{l} u_{2}^{\left(0\right)}\left(x_{1}=m_{8}^{-}\right)=u_{2}^{\left(0\right)}\left(x_{1}=m_{8}^{+}\right)\\ u_{3}^{\left(0\right)}\left(x_{1}=m_{8}^{-}\right)=u_{3}^{\left(0\right)}\left(x_{1}=m_{8}^{+}\right)\\ u_{1}^{\left(1\right)}\left(x_{1}=m_{8}^{-}\right)=u_{1}^{\left(1\right)}\left(x_{1}=m_{8}^{+}\right)\\ T_{5}^{\left(0\right)}\left(x_{1}=m_{8}^{-}\right)=T_{5}^{\left(0\right)}\left(x_{1}=m_{8}^{+}\right)\\ T_{6}^{\left(0\right)}\left(x_{1}=m_{8}^{-}\right)=T_{6}^{\left(0\right)}\left(x_{1}=m_{8}^{+}\right)\\ T_{1}^{\left(1\right)}\left(x_{1}=m_{8}^{-}\right)=T_{1}^{\left(1\right)}\left(x_{1}=m_{8}^{+}\right)\\ \phi^{\left(0\right)}\left(x_{1}=m_{8}^{-}\right)=Ve^{i\omega t} \end{array}$$

Substitution of (13), (17) to (19)–(28) results in 64 non-homogeneous linear equations, then 64 undetermined constants can be solved. The charge $Q_{e}$ on the electrode and the motion capacitance *C* could be obtained as

(29) $$\begin{array}{l} Q_{e}=-D_{3}^{\left(0\right)}\left(x_{3}=j\right)\cdot 2w,\\ C=\frac{Q_{e}}{2V},\\ C_{0}=4\varepsilon_{33}hw/\left(2L\right), \end{array}$$

where $C_{0}$ is the static capacitance. The curve of $C/C_{0}$ with respect to the frequency could be used to determine the resonance modes.

## 4. Results and Discussion

### 4.1. Resonance Modes

According to the theoretical model established above, the forced vibration analysis of the device is carried out through an example. Structural parameters of the array device are shown in Table 1 below.

**Figure 4:**
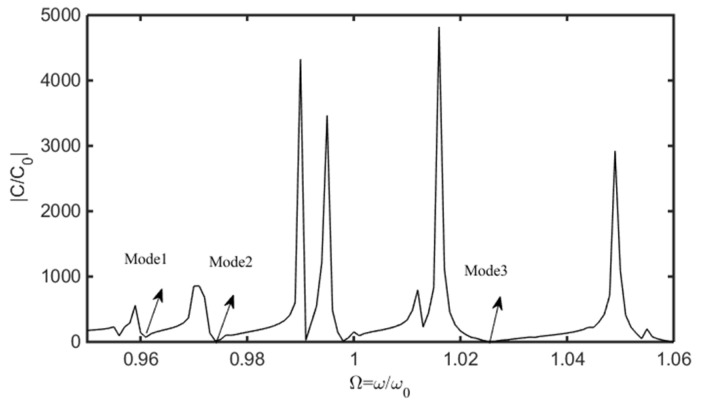
The relationship between the normalized frequency and capacitance ratio of the device.

**Figure 5:**
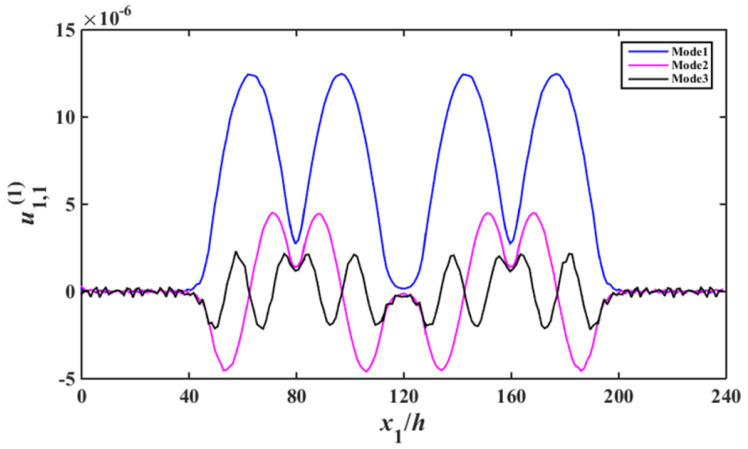
Thickness–twist strain distribution near resonance ($u_{1,1}^{\left(1\right)}$).

**Figure 6:**
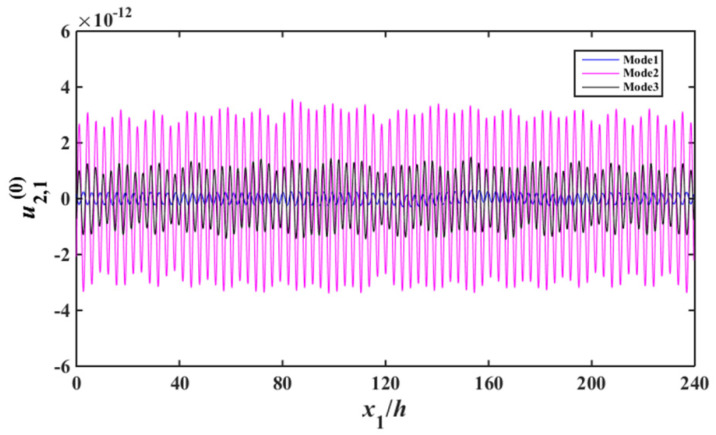
Flexure strain distribution near resonance ($u_{2,1}^{\left(0\right)}$).

**Figure 7:**
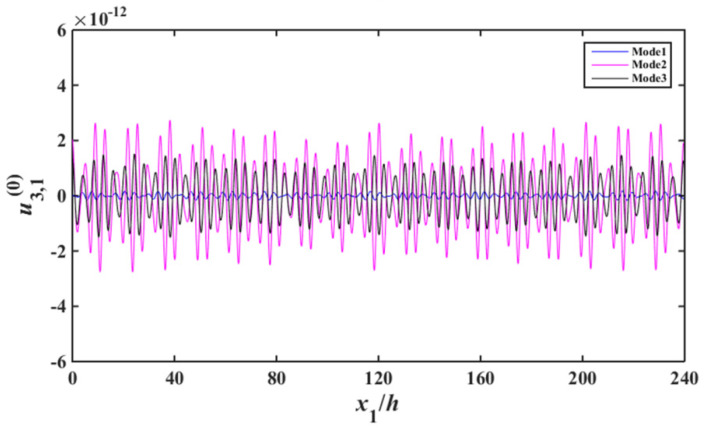
Face-shear strain distribution near resonance ($u_{3,1}^{\left(0\right)}$).

Substituting of (13), (17) to (29) results the according non-homogeneous linear equations, based on which the capacitance ratio vs. frequency of the device are obtained, which is shown in Figure 4. In Figure 4, the abscissa and ordinate are the normalized resonance frequency and the absolute value of capacitance ratio of the device, respectively. For Mode 1, Mode 2, and Mode 3, the displacement distribution curves of thickness-shear, bending and face-shear modes are presented in Figure 5, Figure 6 and Figure 7, respectively.

As can be seen from Figure 5, for Mode 1, strong vibrations exist in the electroded regions of the two resonator units, and the vibrations become weaker obviously in the unelectroded regions of the two resonant units. In the electroded region, the acoustic wave can transmit normally [7]. When the acoustic wave meet the unelectroded region, the wave number become an imaginary number, thus the amplitude of the acoustic wave decrease exponentially, which is the energy-trapping effect [7]. Although Modes 2–3 also have the energy trapping characteristics, their vibration intensity are obviously lower than that of Mode 1. It is shown in Figure 6, the bending vibration intensity of Mode 2 and Mode 3 is much larger than that of Mode1, namely for Mode 1, parasitic modes can be effectively suppressed. As can be seen from Figure 7, for face-shear mode, the vibration of Mode 1 is very weak, and the vibrations of Mode 2 and Mode 3 are stronger, which meets the requirement of parasitic mode suppression of the device. Therefore, Mode 1 is an ideal operating frequency of the device. There are two units in the array device, the approximate operating mode obtained in this work cannot applied to multi-units devices. However, the method used in this work is suitable for multi-units devices.

### 4.2. Frequency Interferences between Two Resonator Units

When the adsorption mass is increased on RU-B, the change of resonant frequency of RU-A reflects the frequency interference of two units. Theoretically, when two resonant units are far enough apart, the frequency interference approaches zero [15]. This spacing is defined as safety spacing. It is necessary to analyze the influence of electrode parameters on the safe spacing.

**Figure 8:**
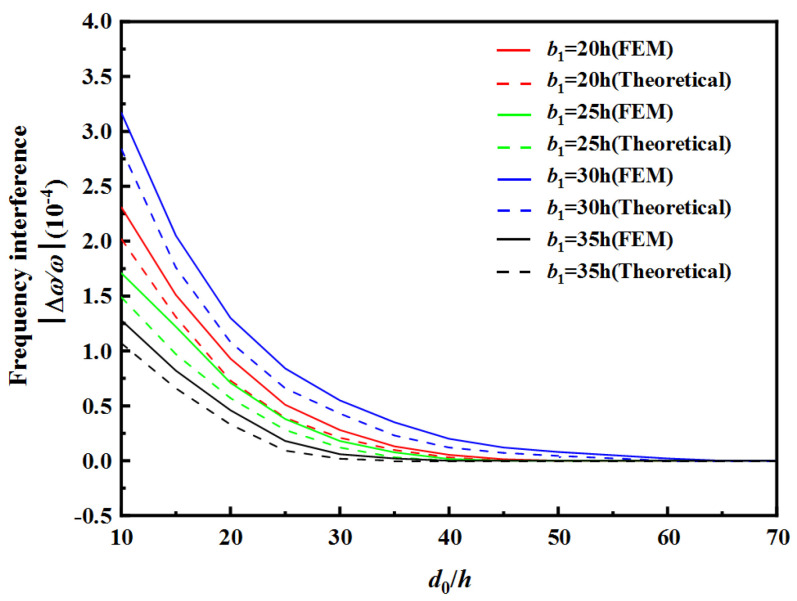
Influences of RU-A’s electrode width on the frequency interference.

**Figure 9:**
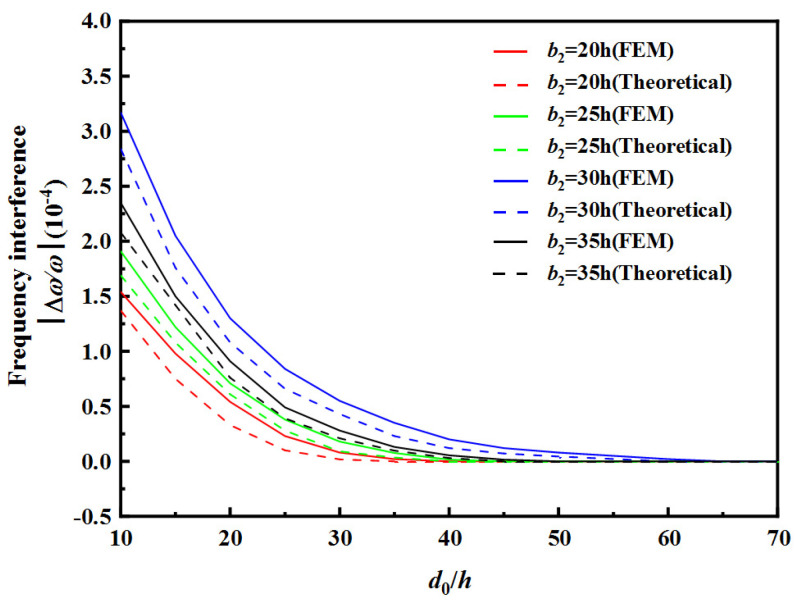
Influences of RU-B’s electrode width on the frequency interference.

Table 2 and Table 3, respectively, show the safe spacing *d*_0_ between two resonator units under different electrode widths. Figure 8 and Figure 9, respectively, show the frequency interference curves when changing the spacing d_0_ between two resonator units under different electrode widths of RU-A and RU-B. Finite element simulation using COMSOL Multiphysics (Burlington, MA, USA), a commercially available modeling package, was performed to obtain the resonance frequency of the array device. This model is a three-dimensional model and the model size parameters are the same as the theoretical model parameters. A frequency domain analysis is carried out to simulate the wave propagation. The calculation results obtained by FEM are slightly higher than the theoretical ones. The observed errors may be due to the differences between the Mindlin plate theory with two-dimensional approximations and the three-dimensional model in the FEM method.

It can be seen from Table 2 and Table 3, as well as Figure 8 and Figure 9, that, with the increase in electrode width of RU-A *b*_1_ or the decrease in electrode width of RU-B *b*_2_, the faster the frequency interference curve tends to 0, the safe distance *d*_0_ between two resonator units also decreases. When the electrode width of RU-A increases, the effective electric field is enhanced, and the vibration intensity of RU-A region is also enhanced, so the anti-interference ability of RU-A becomes stronger. When the electrode width of RU-B decreases, its effect on RU-A is weakened due to the weakening of the effective electric field. When the electrode widths of two resonator unit are equal, the decreasing speed of the frequency interference is smallest, and the safe distance *d*_0_ is maximum.

**Figure 10:**
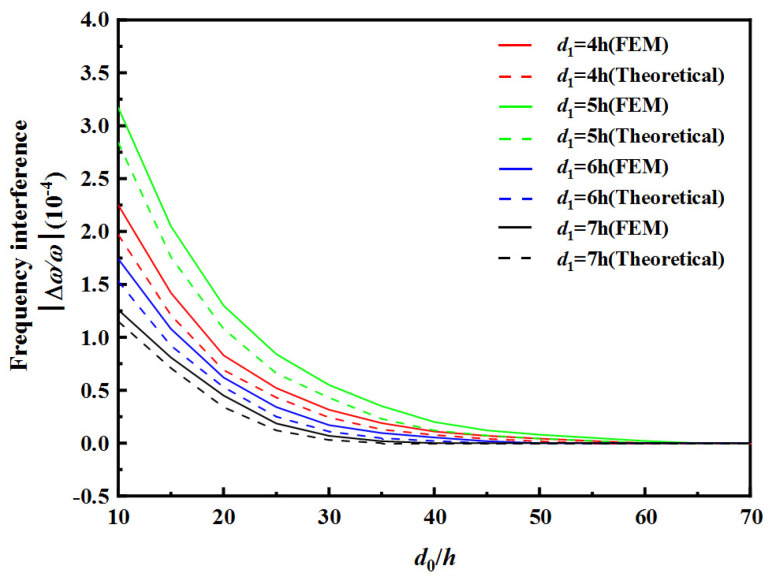
Effect of RU-A’s electrode gap on frequency interference.

**Figure 11:**
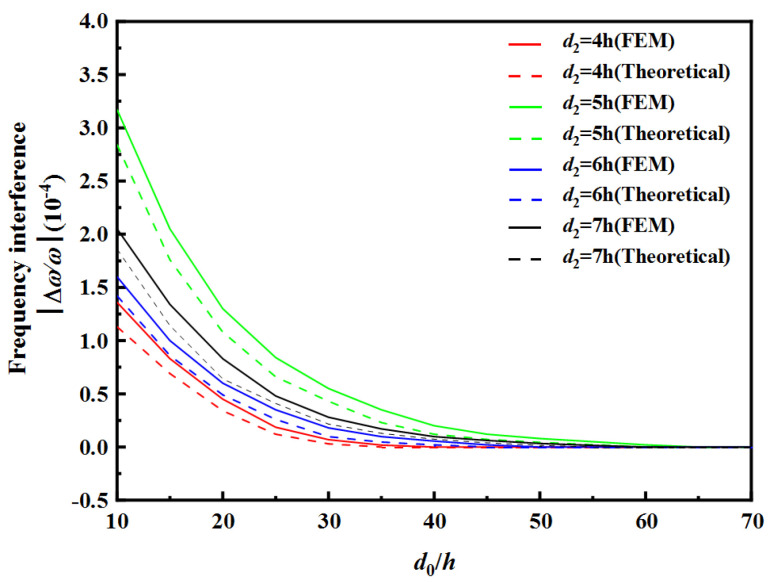
Effect of RU-B’s electrode gap on frequency interference.

Table 4 and Table 5 show the safe spacing *d*_0_ of the two resonator units with different electrode spacing of RU-A *d*_1_ and electrode spacing of RU-B *d*_2_. Figure 10 and Figure 11, respectively, show the frequency interference curves under different electrode spacings of RU-A and RU-B, respectively.

As can be seen from Table 4 and Figure 10, as the electrode spacing value *d*_1_ of RU-A increases, the safe spacing *d*_0_ between the two resonator units decreases, and the frequency interference curve tends to zero faster. As can be seen from Table 5 and Figure 11, as the electrode spacing value *d*_2_ of RU-B increases, the safe spacing *d*_0_ between the two resonator units increases, and the frequency interference curve tends to zero with a lower speed. When the electrode spacings of two resonator unit are equal, the safe distance *d*_0_ is largest, and the frequency interference curve tends to zero slowest.

## 5. Conclusions

In this paper, a theoretical model for analyzing the high-frequency vibration of the LFE bulk acoustic wave array devices based on 3 m point group crystals excited is established, the coupling relationships between vibration modes are clarified, and the influences of structural parameters on the frequency interference between resonator units are revealed. The following conclusions have been obtained: (1) With the increase in electrode width of RU-A or the decrease in electrode width of RU-B, the faster the frequency interference curve tends to 0, the safe distance *d*_0_ between two resonator units also decreases; (2) when the electrode widths of two resonator units are equal, the decreasing speed of the frequency interference is smallest, and the safe distance *d*_0_ is maximum; (3) as the electrode spacing value of RU-A increases, the safe spacing *d*_0_ between the two resonator units decreases, and the frequency interference curve tends to zero faster; (4) when the electrode spacings of two resonator unit are equal, the safe distance is largest, and the frequency interference curve tends to zero slowest. When the electrode structure parameters of the two units are closer, the resonance frequencies of the two units are more similar, thus the frequency interferences are more obviously. There are two units in the array device, the approximate operating mode obtained in this work cannot applied to multi-units devices. However, the method used in this work is suitable for multi-units devices. The theoretical model proposed in this work can provide reliable theoretical basis for parameter optimization designs of strongly coupled array sensors under lateral-field-excitation.

## Figures and Tables

**Table 1:** Parameter setting.

Parameter	Value	Description
ω	10 MHz	Fundamental frequency
2 *h*	0.01755 mm	Thickness of the crystal plate
*L*	239.6 *h*	Length of the crystal plate
2 *w*	119.8 *h*	Width of the crystal plate
*R*	0.05	Mass ratio (Electrode/crystal)
*b*	30 *h*	Width of the electrode
*d*	5 *h*	Space of the two electrodes
*d*_0_	15 *h*	Space of the two resonator units

**Table 2:** The safe distance $d_{0}$ between the two resonant units under different electrode width *b*_1_ of Ru-A.

$b_{1}$	20 h		25 h		30 h		35 h	
	Theoretical	FEM	Theoretical	FEM	Theoretical	FEM	Theoretical	FEM
$d_{0}$	45 h	50 h	40 h	45 h	60 h	65 h	35 h	40 h

**Table 3:** The safe distance $d_{0}$ between the two resonator units under different electrode width *b*_2_ of Ru-B.

$b_{2}$	20 h		25 h		30 h		35 h	
	Theoretical	FEM	Theoretical	FEM	Theoretical	FEM	Theoretical	FEM
$d_{0}$	35 h	40 h	40 h	45 h	60 h	65 h	45 h	50 h

**Table 4:** The safe distance $d_{0}$ between the two resonator units with different electrode gap of RU-A.

$d_{1}$	4 h		5 h		6 h		7 h	
	Theoretical	FEM	Theoretical	FEM	Theoretical	FEM	Theoretical	FEM
$d_{0}$	55 h	60 h	60 h	65 h	45 h	50 h	35 h	40 h

**Table 5:** The safe distance $d_{0}$ between the two resonator units with different electrode gap of RU-B.

$d_{2}$	4 h		5 h		6 h		7 h	
	Theoretical	FEM	Theoretical	FEM	Theoretical	FEM	Theoretical	FEM
$d_{0}$	35 h	40 h	60 h	65 h	45 h	50 h	55 h	60 h

## References

[1] Liu S.Y., Kao Y.H., Su Y.O., Perng T.P. (1999). In situ monitoring of hydrogen absorption-desorption in Pd and Pd_77_Ag_23_ films by an electrochemical quartz crystal microbalance. J. Alloys Compd..

[2] Shen D.Z., Zhang H.T., Kang Q., Zhang H.J., Yuan D.R. (2006). Oscillating frequency response of a langasite crystal microbalance in liquid phases. Sens. Actuators B.

[3] Lu F., Lee H.P., Lim S.P. (2005). Energy-trapping analysis for the bi-stepped mesa quartz crystal microbalance using the finite element method. Smart Mater. Struct..

[4] Liu N., Yang J.S., Jin F. (2012). Transient thickness-shear vibration of a piezoelectric plate of monoclinic crystals. Int. J. Appl. Electrom..

[5] Hu Y.T., Yang J.S., Zeng Y., Jiang Q.A. (2006). High-sensitivity, dual-plate, thickness-shear mode pressure sensor. IEEE Trans. Ultrason. Ferroelectr. Freq. Contr..

[6] Wang S.H., Shen C.Y., Lin Y.M., Lin J.C. (2016). Piezoelecreic sensor for sensitive determination of metal ions based on the phosphate-modified dendrimer. Smart Mater. Struct..

[7] Liu B., Jiang Q., Xie H.M., Yang J.S. (2011). Energy trapping in high-frequency vibrations of piezoelectric plates with partial mass layers under lateral electric field excitation. Ultrasonics.

[8] Hu Y.H., Freench L.A., Radecsky K., Da Cunha M.P., Millard P., Vetelino J.F. (2004). A lateral field excited liquid acoustic wave sensor. IEEE Trans. Ultrason. Ferroelectr. Freq. Contr..

[9] Yang J.S. (2006). The Mechanics of Piezoelectric Structures.

[10] Hempel U., Lucklum R.L., Hauptmann P., EerNisse E.P., Puccio D., Diaz R.F., Vives A.A. Lateral field excited quartz crystal resonators sensors for determination of acoustic and electrical properties of liquids. Proceedings of the IEEE International Frequency Control Symposium.

[11] Wang W.Y., Zhang C., Zhang Z.T., Liu Y., Feng G.P. (2008). Three operation modes of lateral-field-excited piezoelectric devices. Appl. Phys. Lett..

[12] Borodina I.A., Zaitsev B.D., Teplykh A.A., Shikhabudinov A.M., Kuznetsova I.E. (2015). Array of piezoelectric lateral electric field excited resonators. Ultrasonics.

[13] Latif U., Rohrer A., Lieberzeit P.A., Dickert F.L. (2011). QCM gas phase detection with ceramic materials-VOCs and oil vapors. Anal. Bioanal. Chem..

[14] Itoh A., Ichihashi M. (2011). Separate measurement of the density and viscosity of a liquid using a quartz crystal microbalance based on admittance analysis (QCM-A). Meas. Sci. Technol..

[15] Kalchenko V.I., Koshets I.A., Matsas E.P., Kopylov O.N., Solovyov A., Kazantseva Z.I., Shirshov Y.M. (2002). Calixarene-based QCM sensors array and its response to volatile organic vapours. Mater. Sci. Pol..

[16] Sayin S., Ozbek C., Okur S., Yilmaz M. (2014). Perparation of the ferrocene-substituted 1,3-distal p-tert-butylcalix[4]arene based QCM sensors array and utilization of its gas-sensing affinities. J. Org. Chem..

[17] Winters S., Bernhardt G., Vetelino J.F. (2013). A dual lateral-field-excited bulk acoustic wave sensor array. IEEE Trans. Ultrason. Ferroelectr. Freq. Control..

[18] Vetelino J.F. A lateral field excited acoustic wave sensor platform. Proceedings of the IEEE International Ultrasonics Symposium.

[19] Warner A.W., Onoe M., Coquin G.A. (1967). Determination of elastic and piezoelectric constants for crystals in class (3 m). J. Acoust. Soc. Am..

[20] Ma T.F., Zhang C., Zhang Z.T., Wang W.Y., Feng G.P. Investigation of lateral-field-excitation on LiTaO_3_ single crystal. Proceedings of the 2010 IEEE International Frequency Control Symposium.

